# The effects of dendritic cell-based vaccines in the tumor microenvironment: Impact on myeloid-derived suppressor cells

**DOI:** 10.3389/fimmu.2022.1050484

**Published:** 2022-11-15

**Authors:** María Luisa Sánchez-León, Carlos Jiménez-Cortegana, Gabriel Cabrera, Elba Mónica Vermeulen, Luis de la Cruz-Merino, Victor Sánchez-Margalet

**Affiliations:** ^1^ Department of Medical Biochemistry and Molecular Biology, School of Medicine, University of Seville, Seville, Spain; ^2^ Medical Oncology Service, Virgen Macarena University Hospital, Seville, Spain; ^3^ Department of Laboratory Medicine, Virgen Macarena University Hospital, Seville, Spain; ^4^ Laboratorio de Tecnología Inmunológica, Facultad de Bioquímica y Ciencias Biológicas, Universidad Nacional del Litoral, Santa Fe capital, Argentina; ^5^ Laboratorio de Células Presentadoras de Antígeno y Respuesta Inflamatoria, Instituto de Medicina Experimental (IMEX) - CONICET, Academia Nacional de Medicina, Buenos Aires, Argentina

**Keywords:** dendritic cells, myeloid-derived suppressor cells, vaccines, cancer, immunosuppression

## Abstract

Dendritic cells (DCs) are a heterogenous population of professional antigen presenting cells whose main role is diminished in a variety of malignancies, including cancer, leading to ineffective immune responses. Those mechanisms are inhibited due to the immunosuppressive conditions found in the tumor microenvironment (TME), where myeloid-derived suppressor cells (MDSCs), a heterogeneous population of immature myeloid cells known to play a key role in tumor immunoevasion by inhibiting T-cell responses, are extremely accumulated. In addition, it has been demonstrated that MDSCs not only suppress DC functions, but also their maturation and development within the myeloid linage. Considering that an increased number of DCs as well as the improvement in their functions boost antitumor immunity, DC-based vaccines were developed two decades ago, and promising results have been obtained throughout these years. Therefore, the remodeling of the TME promoted by DC vaccination has also been explored. Here, we aim to review the effectiveness of different DCs-based vaccines in murine models and cancer patients, either alone or synergistically combined with other treatments, being especially focused on their effect on the MDSC population.

## Introduction

The success of current cancer therapies depends on the knowledge of the tumor microenvironment (TME), which is a complex signaling network consisting of tumor, immune and stromal cells, as well as non-cellular components such as exosomes or the extracellular matrix (1). Immune cells such as regulatory T cells (Tregs), tumor-associated macrophages (TAMs) or myeloid-derived suppressor cells (MDSCs) expand systematically and play a key role by inhibiting effector T-cell responses and favoring tumor progression through the release of a variety of factors, such as arginase-1 (ARG-1), reactive oxygen species (ROS), interleukin (IL)-6, IL-10, transforming growth factor (TGF)-β, vascular endothelial growth factor (VEGF), or the expression of different proteins in their surface, including programmed cell death protein 1 (PD-1) or cytotoxic T-lymphocyte antigen 4 (CTLA-4), among many others (2–4).

**Figure 1:**
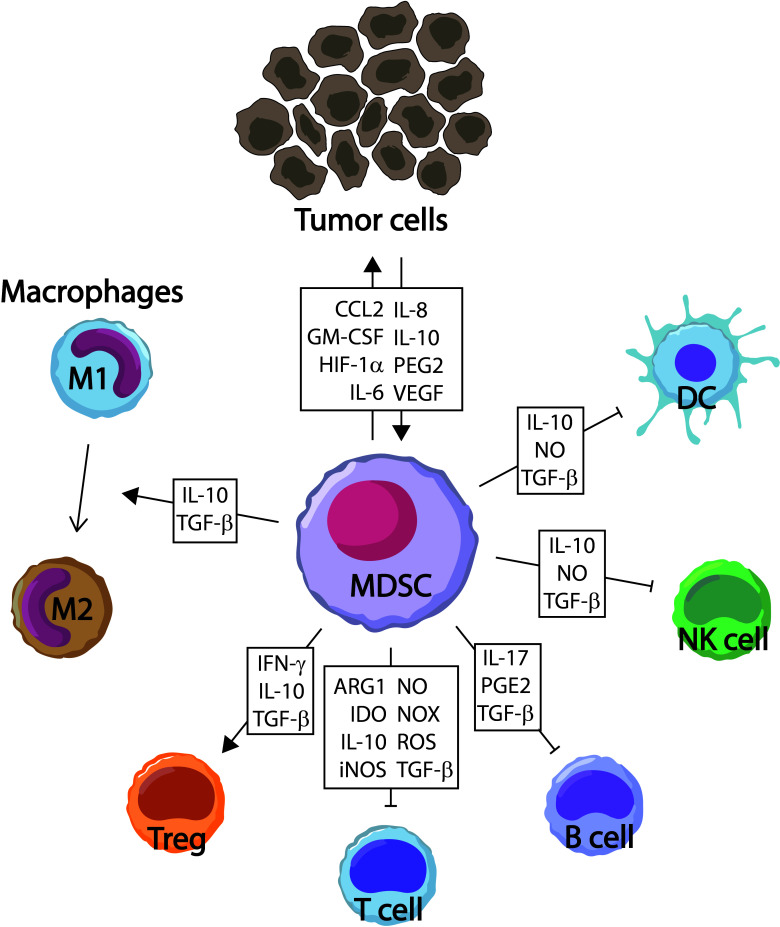
Immunosuppressive role of myeloid-derived suppressor cells (MDSC). MDSC exert their function by promoting the development and expansion of suppressor cells such as M2-macrophages and regulatory T cells (Treg), and inhibiting cell populations that participate in immune responses against the tumor, including dendritic cells (DC), T cells, B cells, and natural killer (NK) cells. Arg, arginase; CCL2, C-C motif ligand 2; GM-CSF, granulocyte/monocyte-colony stimulating factor; HIF, hypoxia-inducible factor; IDO, indoleamine 2,3-dioxygenase; IFN, interferon; IL, interleukin; iNOS, inducible nitric oxide synthase; NO, nitric oxide; NOX, nicotinamide adenine dinucleotide phosphate (NADPH) oxidase; PGE2, prostaglandin E2; ROS, reactive oxygen species; TGF, transforming growth factor; VEGF, vascular endothelial growth factor.

Specifically, MDSCs are a heterogenous population of immature myeloid cells with a potent immunosuppressive capacity (as shown in **Figure 1**) that leads to tumor growth, development of pre-metastatic niches, resistance to immunotherapy, and poor outcomes (5–7). MDSCs are recruited into the TME *via* C-C motif chemokine ligand 2 (CCL2)/C-C motif chemokine receptor 2 (CCR2), CCL3/CCR5, CC15/CCR1, or CXC motif chemokine ligand 13 (CXCL13)/CXC motif receptor 5 (CXCR5) pathways (8), and other mediators such as granulocyte-macrophage colony-stimulating factor (GM-CSF), IL-6, or prostaglandin E2 (PGE2) participate to expand MDSCs. There are two main populations of MDSCs that share some phenotypic, morphological, and functional characteristics with inflammatory, immunosuppressive monocytes and neutrophils, called monocytic MDSCs (M-MDSCs) and granulocytic MDSCs (G-MDSCs) (9). Mouse and human MDSCs share some phenotypic expression, such a CD11b. However, mouse MDSCs are commonly defined as Gr1^+^CD11b^+^ cells characterized by the expression of Ly6C^high^Ly6G^-^ (M-MDSCs) and Ly6C^low^Ly6G^+^ (G-MDSCs), whereas human MDSCs are CD11b^+^HLA-DR^low/-^ cells that also express CD14^+^CD15^-^ (M-MDSCs) or CD14^-^CD15^+^ (G-MDSCs). Of note, G-MDSCs can also express high levels of CD66b (9).

MDSCs mainly inhibit antitumor T-cell responses through a variety of mechanisms (10). In this sense, MDSCs promote the loss of T-cell receptor (TCR) ζ-chain and cell cycle arrest in T cells by up-taking L-cysteine and L-arginine, two essential amino acids for proliferation and expansion of T cells (11, 12). MDSCs release ROS to provoke the loss of the TCR ζ-chain, and promote nitrosylation and nitration of components of the TCR complex (13). MDSCs are also known to downregulate the cytotoxicity of natural killer (NK) cells by releasing TGF-β or indoleamine 2,3-dyoxygenase (IDO) (14). The recruitment of other immunosuppressor cells by releasing IL-10 and TGF-β are also carried out by MDSCs, including M2 macrophages (15) and regulatory T cells (Tregs) (16).

In this context, conventional treatments such as chemotherapy activate multiple signaling pathways and promote the secretion of inflammatory mediators, but it may have dual roles and is not considered as an efficient option to eradicate tumors completely (17), and radiation therapy (RT) causes DNA damage in tumor cells to inhibit their proliferation, but it may also affect to adjacent healthy cells (18). Also, RT, at least under some settings, can promote favorable conditions for immunoevasion by stimulating the recruitment of immunosuppressive cell populations, including MDSCs (19). However, a different approach to treat cancer is based on immunotherapies, which target specific cellular or non-cellular components to boost the potential of the immune system to kill cancer cells. Different immunotherapies have been developed in last decades, including treatments to target stromal cells (20), cell surface proteins (21), or angiogenic factors (22), among others.

Particularly, targeting DC activation using DC-based vaccines can be therapeutically beneficial because DCs are the most potent type of antigen presenting cells and are able to activate their immunogenic machinery *ex profeso* to sample and present tumor-associated antigens (TAAs) to CD4^+^ T cells on major histocompatibility complex (MHC) class II molecules and CD8^+^ T cells on MHC class I molecules, in order to activate T cells to recognize similar TAAs within the TME (23), a process called “cancer-immunity cycle” (24). It has been described that immunogenic tumor cell death (ICD) improves T-cell immunity since it promotes the migration of tumor-infiltrating DCs to draining lymph nodes (25). In addition, ICD determinants have an effect on DCs. For example, proinflammatory mediators of tumor cells such as the high mobility group box 1 (HMGB1) protein or the 70 kilodalton heat shock protein (HSP70) facilitate TAA processing and presentation, whereas plasma membrane components such as calreticulin or phosphatidylserine residues promote phagocytosis or TAA recognition, respectively (26). In line with this, conventional DCs (cDCs) have demonstrated a preferential capacity to promote antigen presentation to T cells (27) rather than monocyte-derived DCs (moDCs) or plasmacytoid DCs (pDCs), which may have dual roles in anti-tumor immunity (28, 29).

Here, we explain the current knowledge regarding the impact of DC-based vaccines on MDSCs in both preclinical tumor models and oncological patients, either alone or combined with other treatments.

## Dendritic cell vaccines and myeloid-derived suppressor cells.

**Figure 2:**
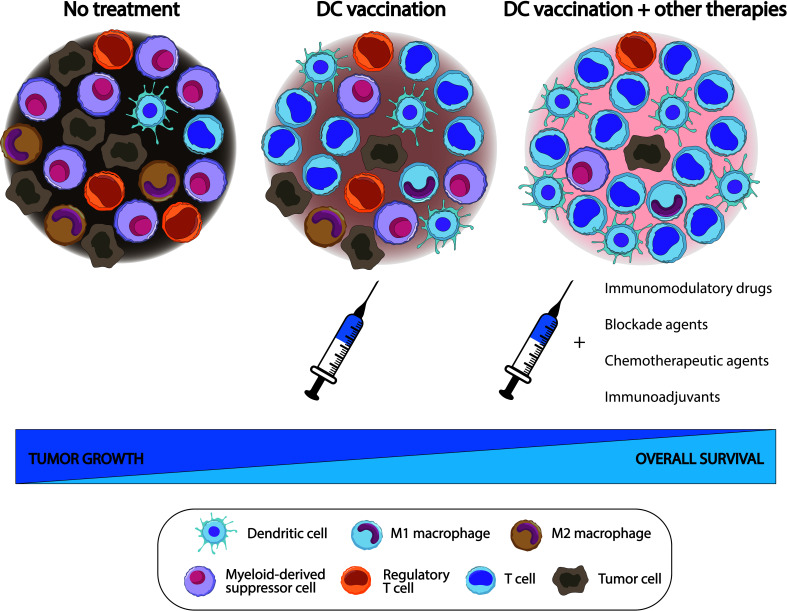
Effects of dendritic cell (DC) vaccination (alone and combined with other treatments) on the tumor microenvironment (TME) in comparison with a non-treated TME. DC vaccines exert a beneficial (although limited) function, increasing the number of lymphoid cell populations within the TME, but with variable levels of myeloid-derived suppressor cells (MDSC), compared with non-treated subjects. However, the addition of some drugs may synergistically potentiate the effects of DC vaccines to further delay tumor growth, improve survival rates, and significantly decrease the number of suppressor cell populations, including MDSC, within the TME.

Immunotherapies have demonstrated to improve outcomes in cancer (30–33). DC infiltration into tumors has been positively correlated with prognosis and survival (34–36), leading to the design of DC-based immunotherapies. DC vaccines emerged as a promising alternative to further improve anti-tumor immunity (37–43). Specifically, cDC vaccines and pDC have shown better anti-tumor efficacy compared to moDC vaccines (44–46). Another interesting approach is the so-called *in vivo* vaccination to target DCs with DC receptor ligands, adjuvants, or other types of molecules that can accurately bind to DCs to exert better anti-tumor responses (47–49). Of note, DC-based immunotherapy could be inefficient due to the MDSC accumulation within the TME, so the combination of DC vaccines with other treatments may be a feasible approach to deplete MDSCs (50, 51), as shown in **Figure 2**.

### Dendritic cell vaccines and myeloid-derived suppressor cells in preclinical tumor models.

The use of DC vaccines has been widely investigated in murine models over the last decades. Although vaccination with DCs has demonstrated effectiveness in certain oncological settings *in vivo* (51–56), it has been extensively shown that the inhibition of immunosuppressive conditions has been slight. In this sense, combinatorial treatments have improved the efficacy of DC vaccines reducing tumor growth significantly, enhancing survival rates, and activating stronger tumor-specific T cell responses (57–62), thus overcoming immune tolerance. A growing preclinical literature illustrates the immunomodulatory capacity of DC vaccines combined with treatments such as IMiDs, inhibitors, or chemotherapeutic agents to reduce the proportion of MDSCs in cancer murine models (**Table 1**).

**Table 1:** Preclinical studies involving the use of dendritic cell-based vaccines (alone and combined with other treatments) and their effects on the myeloid-derived suppressor cell population.

Type of cancer	Tumor model	Type of DC vaccine	Groups of treatment	Effect on MDSCs after treatments	Reference
Breast	4T1 cells	- DC cells - Adenovirus-null DCs - VE-cadherin gene modified DCs	Group 1: control Group 2: DC vaccine Group 3: Adenovirus-null DC vaccine Group 4: VE-cadherin gene modified DC vaccine	Not only MDSCs, but also regulatory T cells, mildly decreased within the tumor tissues of Group 4 compared with the other groups	(63)
		- Tumor lysate-pulsed DCs	Group 1: control Group 2: CD73-specific siRNA-loaded NPs Group 3: DC vaccine Group 4: DC vaccine + CD73-specific siRNA-loaded NPs	Groups 2 and 3 exhibited non-significant reductions of tumor MDSCs, whereas Group 4 showed a remarkable decrease, and their levels were significantly correlated with the frequency of CD73^+^ cells in tumor tissue	(64)
Colon	CT26 cells	- DC cells - Adenovirus-null DCs - VE-cadherin gene modified DCs	Group 1: control Group 2: DC vaccine Group 3: Adenovirus-null DC vaccine Group 4: VE-cadherin gene modified DC vaccine	Not only MDSCs, but also regulatory T cells, mildly decreased within the tumor tissues of Group 4 compared with the other groups	(63)
	MC-38 cells	- Tumor antigen-loaded DCs	Group 1: control Group 2: LEN Group 3: DC vaccine Group 4: LEN + DC vaccine	Group 4 showed the lowest percentage of splenic MDSCs	(54)
		- Tumor antigen-loaded DCs	Group 1: control Group 2: TA-DC vaccine Group 3: Rv2299c Group 4: TA-DC vaccine + Rv2299c	Proportions of MDSCs increased in Group 1, remained unchanged in Groups 2 and 3, and significantly decreased in Group 4	(65)
Glioma	GL261 cells	- Lysate-pulsed mature DCs	Group 1: Control Group 2: freeze-thaw necrosis (FT) + DC vaccine Group 3: FT + radiation + DC vaccine	There was a decrease of both MDSC subsets and TAMs in Group 3 compared to Groups 1 and 2	(66)
Hepatocellular	Hepa1-6 cells	- DC/tumor cell fusion vaccine	Group 1: control Group 2: folate-modified chitosan/mouse interferon-induced protein-10 (FC/MIP10) Group 3: FC/MIP10 + DC vaccine	Group 3 significantly reduced MDSCs in spleen, tumor, and bone marrow and increased tumor-specific IFN-γ responses compared with the other groups.	(67)
Kidney	HEK293 cells	- DC cells - Adenovirus-null DCs - VE-cadherin gene modified DCs	Group 1: control Group 2: DC vaccine Group 3: Adenovirus-null DC vaccine Group 4: VE-cadherin gene modified DC vaccine	Not only MDSCs, but also regulatory T cells, mildly decreased within the tumor tissues of Group 4 compared with the other groups	(63)
Lung	Lewis Lung Carcinoma cells	- Rose Bengal (RB)-immature DCs - RB-mature DCs	Group 1: control Group 2: RB Group 3: RB-immature DC vaccine Group 4: RB- mature DC vaccine	MDSCs significantly decreased in groups 3 and 4 within the tumor microenvironment compared to control groups, whereas MDSC levels remained unchanged in the spleens.	(55)
		- TAA-derived MHC class I peptide- mature DCs	Group 1: healthy Group 2: Control Group 3: DC vaccine Group 4: anti-cancer-associated fibroblasts (CAFs) Group 5: anti-CAFs + DC vaccine	Groups 3, 4, and 5 showed a decrease of MDSCs, but it was significant in Group 5, reaching levels of Group 1	(68)
Lymphoma	A20 B cells	- CFSE-labeled DCs	Group 1: control Group 2: Gemcitabine Group 3: DC vaccine Group 4: Gemcitabine + DC vaccine	Groups 2 and 3 did not inhibit tumor growth. However, the addition of gemcitabine to the vaccine (Group 4) significantly reduced MDSCs and improved efficacy	(51)
	E.G7 cells	- TAA-derived MHC class I peptide- mature DCs	Group 1: healthy Group 2: Control Group 3: DC vaccine Group 4: anti-CAFs Group 5: anti-CAFs + DC vaccine	Groups 3, 4, and 5 showed a decrease of MDSCs, but it was significant in Group 5, reaching levels of Group 1	(68)
Melanoma	B16F10 cells	- Mature DCs	Group 1: control Group 2: DC vaccine Group 3: low-dose 5-fluorouracil (5FU) Group 4: DC vaccine + low-dose 5FU	MDSCs similarly decreased in groups 2, 3 and 4 compared with the control group.	(69)
		- Tyrosinase related protein (TRP)-1/Tyrosine (Tyr) DCs	Group 1: DC vaccine Group 2: DC vaccine + paclitaxel Group 3: DC vaccine + anti-PD-1	Groups 2 and 3 experienced stronger cytotoxic T-cell activation and significantly decreased MDSCs in tumor-bearing mice, which led to improved survival rates	(70)
		- Mature DCs	Group 1: control Group 2: DC vaccine Group 3: low-dose 5FU Group 4: DC vaccine + low-dose 5FU	Group 4 showed a reduced number of MDSCs and tumor growth, as well as increased survival, compared with the other groups	(50)
	B16F1 cells	- TAA-derived MHC class I peptide- mature DCs -	Group 1: healthy Group 2: Control Group 3: DC vaccine Group 4: anti-CAFs Group 5: anti-CAFs + DC vaccine	Groups 3, 4, and 5 showed a decrease of MDSCs, but it was significant in Group 5, reaching levels of Group 1	(68)
	B16.OVA cells	- OVA peptide-pulsed DC.IL12 cells	Group 1: Control Group 2: DC vaccine Group 3: Dasatinib Group 4: Dasatinib + DC vaccine	MDSCs were especially depleted in Group 4, which was associated with a reduction of hypoxic signalling	(56)
	MO5-B16 cells	- Tyrosinase related protein (TRP)-1/Tyrosine (Tyr) DCs	Group 1: DC vaccine Group 2: DC vaccine + paclitaxel Group 3: DC vaccine + anti-PD-1	Groups 2 and 3 experienced stronger cytotoxic T-cell activation and significantly decreased MDSCs in tumor-bearing mice, which led to improved survival rates	(70)
Myeloma	MOPC-315 cells	- Dying tumor cell-loaded DCs	Group 1: control Group 2: DC vaccine Group 3: pomalidomide + dexamethasone (POM/DEX) Group 4: DC vaccine + POM/DEX	Group 4 exhibited the lowest generation of splenic MDSCs, which was associated with a greater inhibition of tumor growth	(71)
		- Dying tumor cell-loaded DCs	Group 1: control Group 2: DC vaccine + POM/DEX Group 3: POM/DEX + anti-PD-1 Group 4: DC vaccine + POM/DEX + anti-PD-1	Pomalidomide with dexamethasone + PD-L1 from Groups 3 and 4 decreased the generation of MDSCs and Tregs in both the spleen and TME compared to Groups 1 and 2	(53)
		- Dying tumor cell-loaded DCs	Group 1: control Group 2: DC vaccine Group 3: DC vaccine + lenalidomide (LEN) Group 4: DC vaccine + anti-PD-1 Group 5: DC vaccine + LEN + anti-PD-1	Splenic MDSCs were dramatically reduced in all treatment groups compared to control, but Group 5 showed the lowest proportion of these cells	(52)
	YAC-1 cells	- Dying tumor cell-loaded DCs	Group 1: control Group 2: DC vaccine + POM/DEX Group 3: POM/DEX + anti-PD-1 Group 4: DC vaccine + POM/DEX + anti-PD-1	Pomalidomide with dexamethasone + PD-L1 from Groups 3 and 4 decreased the generation of MDSCs and Tregs in both the spleen and TME compared to Groups 1 and 2	(53)
Pancreatic	UNKC6141 PaCa cells	- Tumor cell-derived exosomes-loaded DCs	Group 1: control Group 2: Gemcitabine, ATRA, Sunitinib (GAS) Group 3: tumor exosome-loaded (TEX) DC vaccine Group 4: GAS + TEX DC vaccine	Group 2 experienced a significant reduction of both MDSCs, and tumor cells compared with the other groups. However, Group 3 and 4 prolonged the survival time, but persisting drug application promoted tumor reappearance in the last group.	(72)
Pancreatic	Panc02 cells	- Mature DCs	Group 1: control Group 2: Gemcitabine Group 3: DC vaccine Group 4: Gemcitabine + DC vaccine	Gemcitabine decreased MDSCs in spleens and tumors of Group 2. Its addition to DC vaccination (Group 4) also improved survival rates in mice	(73)

#### IMiDs

Lenalidomide (LEN) and pomalidomide (POM) are IMiDs derived from thalidomide that increase cytotoxic responses driven by T cells and NK cells against tumors (74, 75). LEN has promoted the depletion of MDSCs in lymphoma patients with good response to the treatment (76) and in lymphoma-bearing mice, in which have been demonstrated an additive therapeutic antitumor effect when combined with a fusion DNA vaccine (77). In combination with TAA-loaded DC vaccine, LEN showed a remarkable tumor growth inhibition and significantly reduced MDSCs compared to LEN alone and DC vaccine alone in a colon mouse model (54).

Promising results were obtained with POM combined with dexamethasone in multiple myeloma (MM) (78, 79), although the combination with different inhibitors could further improve cell cycle arrest, deregulation of metabolic pathways, and tumor cell apoptosis in proliferative phases(80–82). When added to a DC vaccine, POM and dexamethasone synergistically improved antitumor immunity in MM mouse models due to the increased proportion of effector lymphoid cells and the depletion of not only splenic MDSCs, but also VEGF (71), which usually promotes angiogenesis and MDSC migration into the blood (83, 84).

#### Inhibitors

The use of blockade agents has also improved the efficacy of different types of vaccines (85, 86). Specifically, immune checkpoint inhibitors have successfully targeted MDSCs in melanoma-bearing mice treated with the tyrosinase related protein (TRP)1/tyrosine DC vaccine (70). Similar results were obtained in myeloma murine models after combining a DC vaccine and anti-PD-1 with IMiDs (52, 53).

In addition, DC vaccines has improved the effects of other inhibitors such as tranilast (TRA) or dasatinib (DAS) in lung and melanoma mouse models, respectively, because the combinatorial treatment reduced the number of MDSCs (56, 68). TRA is an anti-fibrotic agent to inhibit not only cancer-associated fibroblasts (CAFs; which are one of the most abundant and critical components of the tumor mesenchyme to promote carcinogenesis), but also tumor cell interactions, and the modulation of immune factors (87, 88), including MDSC differentiation and recruitment (89, 90). In the same line, DAS allows the blockade of the SRC kinase family (91) and has shown to improve T-cell responses after decreasing MDSCs in head and neck squamous cell carcinoma (92). Of note, M-MDSCs have been suggested as a promising prognostic biomarker in patients with myeloid leukemia treated with DAS (93).

#### Antimetabolite drugs

Analogs of biological compounds to inhibit metabolic routes also reduced the proportion of MDSCs when combined with DC-based vaccines. 5-fluorouracil (5-FU) is a fluoropyrimidine that inhibits essential biosynthetic processes and RNA and DNA functions by incorporating their metabolites into the nucleic acids and inhibiting the enzyme thymidylate synthase (94), which promotes the depletion of MDSCs (95). 5-FU in combination with a DC vaccine showed a greater MDSC reduction and improved survival rates in melanoma-bearing mice compared with DC vaccination alone (which maintained MDSC levels), and/or 5-FU alone (which significantly depleted MDSCs, although survival rates were lower compared to the combinatorial treatment) (50). Interestingly, these schedules were used in a melanoma-bearing mice to stablish an agent-based model to simulate the interactions between tumor and immune cells, as well as comparing different scenarios to determine the role of each component (including MDSCs) during tumor progression (69).

Also, rose bengal (RB), a staining agent and an inhibitor of ribonucleic acid chain elongation some decades ago (96), induced not only the regression of injected tumors in melanoma murine models, but also immunogenic cell death and the release of HMGB1, which improved DC infiltration into draining lymph nodes and, consequently, the activation of T cell responses (97). Combined with a DC vaccine, RB reduced MDSCs and enhanced the activation of effector cells and the release of TNF-α, leading to the inhibition of tumor growth in lung cancer-bearing mice (55).

#### Chemotherapeutic agents

DC vaccines have also been combined with chemotherapeutic agents such as gemcitabine (GEM). GEM is known to inhibit DNA synthesis of tumor cells (98) and has shown to inhibit MDSC expansion both *in vitro* and *in vivo*  *(* 99–101). In line with this notion, DC vaccination improved the effects of low-dose GEM due to not only DC maturation, T-cell activation, and the production of IFN-γ, but also the reduction of tumor cells and MDSCs (GEM alone also did), and tumor growth inhibition, thus enhancing survival rates (GEM alone did not) in lymphoma-bearing mice (51). GEM alone increased the apoptosis of splenic MDSCs significantly in pancreatic cancer-bearing mice, whereas the combination with a DC vaccine also enhanced the overall survival (73).

In a pancreatic cancer mouse model, overall survival was prolonged after using a tumor-exosome (TEX)-DC vaccine but both circulating and tumor-infiltrating MDSCs were only depleted in the group of mice treated with GEM, all-trans retinoic acid (ATRA), and/or sunitinib, or the combinatorial schedule with the vaccine. However, the latter group unexpectedly experienced tumor reappearance due to persisting drug application (72).

#### Other combinatorial treatments

Chitosan is a biological polysaccharide that play a role in multiple medical functions, such as absorption enhancer or drug releaser. In cancer, chitosan is mainly used in chemotherapeutic delivery and as an immunoadjuvant in vaccines (102). Folate (FA)-modified chitosan has demonstrated to enhance tumor targeting and cytotoxic T-cell responses in some oncological settings (103–106). FA-modified chitosan nanoparticles carrying a plasmid of the mouse interferon-induced protein-10 (mIP-10) gene, a chemoattractant for cytotoxic T cells, reduced MDSCs from spleen, tumor, and bone marrow in combination with a DC/tumor cell fusion vaccine in hepatocellular carcinoma-bearing mice (67). In this context, neovascularization, metastasis, and survival of cancer cells can also be promoted by adenosine and its producing molecules, such as CD73, which has been proposed as one of the next-generation targets in cancer (107, 108). In line with this notion, CD73 expression is a biomarker of poor prognosis in breast cancer (109) and the use of chitosan-lactate nanoparticles to target CD73-specific small interfering RNA (siRNA) in combination with a DC vaccine have been successfully tested in breast cancer models after inhibiting tumor growth, and MDSCs (64). Interestingly, this combinatorial treatment also reduced the levels of matrix metalloproteinase (MMP)-2 and MMP-9, which are pro-angiogenic factors released by MDSCs to facilitate extravasation processes and angiogenesis (110).

In addition, vascular endothelial (VE)-cadherin is an adhesion molecule with a key role in the development of the blood vascular system (111). VE-cadherin promotes tumor development and progression by enhancing angiogenesis (112) *via* interaction with VEGF receptor-2 and stimulation of TGF-β signaling pathway (113, 114). For these reasons, a VE-cadherin gene modified DC-based vaccine was developed and successfully tested in kidney, breast, and colon cancer models, resulting in delayed tumor progression and enhancing survival rates by the production of a large amounts of immunoglobulins, and the increase of T effector cells and cytotoxicity against VE-cadherin, as well as the reduction of immunosuppressor cells, including MDSCs (63).

The protein Rv2299c has also demonstrated to induce DC activation and maturation, and enhance the expression of MHC molecules, CD80, and CD86 proteins *in vitro* to promote naïve-T-cell proliferation (115). Combined with a DC vaccine, Rv2299c reduced tumor growth in a colon cancer murine model and improved antitumor immunity by activating T-cell responses and reducing MDSCs (65).

Interestingly, a DC vaccine with irradiated freeze-thaw-necrotic cells has also been tested and it increased tumor rejection and reduced the number of immunosuppressive cells, including MDSCs (66).

### Dendritic cell vaccines and myeloid-derived suppressor cells in cancer patients.

Immunotherapy consisting in DC vaccination alone or combined with other treatments has also been tested in a variety of clinical trials (116–123). Most of them has been focused on drug effectiveness and efficacy rather than the analysis of immunosuppressive cell populations. Therefore, the impact of DC vaccination on human MDSCs (**Table 2**) has been little studied.

**Table 2:** Clinical studies involving the use of dendritic cell-based vaccines (alone and combined with other treatments) and their effects on the myeloid-derived suppressor cell population.

Type of cancer	Type of DC vaccine	Groups of treatment	Effect on MDSCs after treatments	Reference
Brain	GBM6-AD/DCs	DC vaccine	Patients with stable disease experienced a significant decrease of early stage MDSCs and M-MDSCs. Also, a significant increase of G-MDSCs in patients with non-stable disease was observed.	(124)
Breast	Monocyte-derived autologous DCs	Group 1: Control Group 2: DC vaccine + neoadjuvant chemotherapy (NAC)	MDSCs significantly decreased in Group 2 after treatment	(125)
Breast	Wilms tumor gene (WT) 1 peptide-pulsed DCs	DC vaccine+ NAC	Patients who had immunological response to treatment had a significant depletion of MDSCs	(126)
Esophageal	WT1 peptide-pulsed DCs	DC vaccine + docetaxel	Positive immune responses were significantly correlated with a low concentration of MDSCs	(127)
Gastric	WT1 peptide-pulsed DCs	DC vaccine+ NAC	Patients who had immunological response to treatment had a significant depletion of MDSCs	(126)
Lung	Wild-type p53-transduced DCs	Group 1: Control Group 2: DC vaccine Group 3: DC vaccine + ATRA	MDSC levels were similar in all groups before starting treatments. After vaccinations, MDSCs did not vary in Group 2, whereas they decreased more than two-fold in Group 3	(128)
Melanoma	Tumor-associated antigen (TAA)-loaded monocyte-derived autologous DCs	Group 1: Healthy control Group 2: DC vaccine	M-MDSCs were significantly higher in Group 2 compared to Group 1. After treatment, MDSC frequency was associated with short survival.	(129)
Melanoma	AdVTMM2-transduced DCs	Group 1: Healthy control Group 2: DC vaccine Group 3: DC vaccine + IFNα	In group 2, there was a decreased of HLA-DR^−^CD11b^+^CD33^+^ MDSCs, although M-MDSCs and G-MDSCs were not reduced. Group 3 experienced a slightly decreased of MDSC subsets	(130)
Ovarian	WT1 peptide-pulsed DCs	DC vaccine+ NAC	Patients who had immunological response to treatment had a significant depletion of MDSCs	(126)
Prostate	Autologous DCs	Group 1: Docetaxel Group 2: Docetaxel + DC vaccine	MDSC levels were similar before treatments in both groups. However, Group 2 had a significant MDSC decrease during treatment compared to Group 1	(131)
Renal	Tumor lysate-loaded DC	DC vaccine + sunitinib	Patients who responded to treatment showed decreased levels of MDSCs, whereas those who failed to develop tumor-reactive T cell responses did not show consistent reductions of MDSCs	(132)
Sarcoma	TAA-monocyte-derived DCs	DC vaccine	MDSC levels varied in every patient after vaccination. One of those patients had low levels of M-MDSCs and experienced a remarkable regression of metastatic lesions	(133)

DC vaccines alone have shown conflicting results in terms of MDSC levels. MoDC vaccines loaded with TAAs demonstrated the stimulation of a preexisting immune response against TAAs in children, adolescents, and young adults with sarcoma tumors in a phase I/II clinical trial. Interestingly, one of those patients had low levels of MDSCs and Tregs prior to vaccination and experienced significant regression of metastatic lesions after a second disease relapse (133). A similar vaccine was tested in a clinical trial involving metastatic melanoma patients, but M-MDSCs increased after vaccination and were inversely associated with survival (129), demonstrating their immunosuppressive role.

Another anti-tumor target is the stromal tumor suppressor p53, since its loss showed to modify cytokine secretion to increase myeloid infiltration (134), including MDSCs (135). In this sense, the deletion of stromal p53 demonstrated to increase the proliferation of fibroblasts and epithelial cells in KrasG12D-bearing mammary glands together with DNA damage and replication stress, which finally reduced apoptosis of tumor cells and increased the frequency of MDSCs (136). The DC vaccine transduced with wild-type p53 was tested in small cell lung cancer patients without previous positive p53 responses and only 20% of them developed p53-specific responses, with no significant variations in the levels of granzyme B-positive CD8 T cells and MDSCs (128).

In addition, promising results have been shown after using a adenovirus (AdV)-loaded DC vaccine with the TAAs tyrosinase, melanoma-associated antigen recognized by T cells (MART-1), and melanoma-antigen gene (MAGE)-A6 (collectively known as TMM2) since it produced the depletion of blood HLA-DR^−^CD11b^+^CD33^+^ MDSCs (130). Even better, a DC-based vaccine loaded with the glioma stem cell line GBM6*-*AD promoted a significant decrease of both M-MDSCs and e-MDSCs in patients with stable disease, whereas their non-stable counterparts experienced an increase of G-MDSCs (124).

Conversely, DC-based vaccines combined with other treatments have notably improved clinical responses and were associated with a significant MDSC reduction compared to DC vaccination alone:

#### Targeted therapies

*IFNα*, which belongs to the IFN1 family, is downregulated in the MDSC gene expression profile (137), suggesting that IFN-α signaling may be a key pathway to restrict the suppressive activity of MDSCs (138). Also, nitric oxygen produced by MDSCs may reduce the IFN responsiveness in other immune cells *in vivo* (139). Therefore, IFN-α therapies began to be tested, demonstrating an increment of the antibody-dependent cellular cytotoxicity in tumor-bearing mice (140), but adverse effects were also reported and they should be taken into consideration (141). When combined with an AdVTMM2-transduced DC vaccine in melanoma patients, IFN-α slightly decreased both M-MDSCs and G-MDSCs compared to DC vaccination alone and did not improve clinical responses (130).

Contrarily, sunitinib, which is a multi-target tyrosine kinase inhibitor that blocks stem cell factor receptor c-KIT, platelet-derived growth factor receptors, CSF receptors, and VEGF receptors 1, 2 and 3, has been extensively described as a treatment for renal cell carcinoma (142–144). Sunitinib reduced the level of MDSCs (145, 146), mainly due to the inhibition of the signal transducer and activator of transcription (STAT)3 signaling pathway (147). TAA-loaded DC-based vaccine improved the effects of sunitinib due to the reduction in the percentage of MDSCs in patients with renal cell carcinoma (132).

Another compound, ATRA, which is a targeted therapy for peptidylprolyl cis/trans isomerase, NIMA-Interacting 1 (best known as Pin1) in breast cancer and acute promyelocytic leukemia (148), is an active metabolite of vitamin A and exerts important effects not only in cell growth, differentiation, and apoptosis (149), but also in enhancing the influx of DCs into the draining lymph nodes (150) and promoting the maturation of MDSCs into neutrophils and monocytes (151). Specifically, vaccination with p53-transduced DCs combined with ATRA in lung cancer patients resulted in a significant depletion of MDSCs in more than twofold, that was accompanied by more p53-specific responses compared to vaccination alone (128).

#### Chemotherapeutic agents

When combined with vaccines, chemotherapeutic agents may be promising strategies (152) because they improve the efficacy of DC vaccination synergistically in cancer patients (153, 154). Neoadjuvant chemotherapy (NAC) demonstrated to increase the levels of MDSCs in breast cancer (155, 156), whereas DC vaccination with conventional NAC was suggested to be more effective in cancer patients with lower MDSCs and Tregs prior to treatment because they may develop beneficial immunological responses (126). However, other studies have reported pathological complete responses after depleting MDSCs in breast cancer patients (157, 158), similarly to the combination with an autologous Mo-DC vaccine (125).

Also, the chemotherapeutic drug docetaxel, which has shown to boost immune responses after inducing M1 macrophages and antigen presentation *in vitro* (159) and inhibiting MDSCs in breast cancer *in vivo* (160), depleted MDSCs and promoted positive immune responses when combined with DC vaccines in esophageal and prostate cancers (127, 131).

## Conclusions and future perspectives

DC vaccines emerged two decades ago as alternative strategies to overcome tumor resistance and improve survival rates in cancer. DC vaccination has demonstrated better (although still limited) results in terms of efficacy and overall survival compared with other treatments. Probably, cellular immune responses promoted by DC vaccines alone are not sufficiently strong to overcome immunosuppression. Despite this, DC vaccines are currently considered as promising therapeutic approaches to be still optimized. In this sense, the addition of different drugs may be needed to synergically improved the effects of this type of vaccination, what should be further tested in both murine models and clinical trials.

Tumor resistance is mainly led by cells with suppressive functions. Specifically, MDSCs are considered as the “queen-bee” cell population of the TME because they lead multiple mechanisms of resistance and finally protect tumor cells for their surveillance and proliferation (161). In this situation, a variety of therapeutic approaches are currently being used to target MDSCs in cancer, such as their direct elimination, preventing their recruitment into the TME, inhibiting their immunosuppressive role, or inducing their differentiation into mature myeloid cells (162).

The therapeutic benefit of DC vaccines as monotherapies may have been limited due to the resistance promoted by MDSCs, which is supported by the extensive literature that address the conflicting results regarding the levels of MDSCs after using DC vaccines. However, combinatorial approaches with other drugs such as IMiDs, antimetabolites, or chemotherapeutic agents have shown to reduce tumor growth and improve overall survivals *in vivo* compared to DC vaccines alone, which have been also associated with a significant depletion of MDSCs in clinical trials. Therefore, it seems clear that targeting MDSCs with combinatorial regimens based on DC vaccination plus different types of treatment may be therapeutically useful. However, we should take into consideration some aspects, including (a) the type of DCs used in the vaccine (e.g., cDCs can initiate effective immune responses, whereas moDCs seems to play a dual role in cancer) (b), the type of adjuvant (e.g., some vaccines have been loaded with TAAs, but others with dying tumor cells or RB) (c), the dosage used, that may depends on the status of the patients (e.g. levels of blood lymphocytes or MDSCs) and may be personalized, and (d) adverse effects (e.g., as it is with the use of IFN-α therapies). Altogether, some therapeutic approaches to overcome MDSC-mediated immunosuppression and improve survival rates may be focused on DC vaccination combined with MDSC inhibitors (e.g., receptor antagonists of cytokines or chemokines that recruit MDSC into the tumor), which may be promising, alternative strategies to be tested *in vitro* and *in vivo* in the future.

## Author contributions

MLS-L, C-J-C, GC, MV, LC-M, and VS-M contributed to conceptualization, literature search, and reviewing of the draft. MLS-L, and CJ-C wrote the draft. All authors have read and agreed to publish this version of the manuscript.

## Funding

CJ-C is supported by a Margarita Salas fellowship, granted by the University of Seville (Seville, Spain).

## Conflict of interest

The authors declare that the research was conducted in the absence of any commercial or financial relationships that could be construed as a potential conflict of interest.

## Publisher’s note

All claims expressed in this article are solely those of the authors and do not necessarily represent those of their affiliated organizations, or those of the publisher, the editors and the reviewers. Any product that may be evaluated in this article, or claim that may be made by its manufacturer, is not guaranteed or endorsed by the publisher.
